# Surface Functionalization of Magnetite Nanoparticles with Multipotent Antioxidant as Potential Magnetic Nanoantioxidants and Antimicrobial Agents

**DOI:** 10.3390/molecules27030789

**Published:** 2022-01-25

**Authors:** Syed Tawab Shah, Zaira Zaman Chowdhury, Mohd. Rafie Bin Johan, Irfan Anjum Badruddin, H. M. T. Khaleed, Sarfaraz Kamangar, Hussein Alrobei

**Affiliations:** 1 Nanotechnology and Catalysis Research Center, University of Malaya, Kuala Lumpur 50603, Malaysia; tawab_shah2003@yahoo.com (S.T.S.); mrafiej@um.edu.my (M.R.B.J.); 2 Department of Mechanical Engineering, College of Engineering, King Khalid University, Abha 61421, Saudi Arabia; irfan@kku.edu.sa (I.A.B.); shiblygsa@gmail.com (S.K.); 3 Research Center for Advanced Materials Science (RCAMS), King Khalid University, Abha 61413, Saudi Arabia; 4 Department of Mechanical Engineering, Faculty of Engineering, Islamic University of Madinah, Medina 42351, Saudi Arabia; khalid_tan@yahoo.com; 5 Department of Mechanical Engineering, Faculty of Engineering, Prince Sattam Bin Abdulaziz University, Al-Kharj 16278, Saudi Arabia; h.alrobei@psau.edu.sa

**Keywords:** functionalization, magnetite nanoparticles, nanoantioxiants

## Abstract

Functionalized magnetite nanoparticles (Fe_3_O_4_) were prepared using the coprecipitation method followed by functionalization with a multipotent antioxidant (MPAO). The MPAO was synthesized and analyzed using FTIR and NMR techniques. In this study, the functionalized nanoparticles (IONP@AO) were produced and evaluated using the FTIR, XRD, Raman, HRTEM, FESEM, VSM, and EDX techniques. The average determined particle size of IONP@AO was 10 nanometers. In addition, it demonstrated superparamagnetic properties. The magnitude of saturation magnetization value attained was 45 emu g^−1^. Virtual screenings of the MPAO’s potential bioactivities and safety profile were performed using PASS analysis and ADMET studies before the synthesis step. For the DPPH test, IONP@AO was found to have a four-fold greater ability to scavenge free radicals than unfunctional IONP. The antimicrobial properties of IONP@AO were also demonstrated against a variety of bacteria and fungi. The interaction of developed nanoantioxiants with biomolecules makes it a broad-spectrum candidate in biomedicine and nanomedicine.

## 1. Introduction

Antioxidants, recognized as prophylactic and therapeutic molecules, have various applications in the field of pharmaceuticals, cosmetics and nutraceuticals due to the many health benefits associated with their usage [1]. Further study is being done to better understand the involvement of antioxidants in the redox biological pathway and to strengthen their ability to protect cells from reactive oxygen species (ROS). The phrase ‘oxidative stress’ refers to an imbalance between the production of ROS and the body’s response to these ROS. Internally generated ROS damage proteins, DNA, and lipids permanently cause genetic mutations and ultimately lead to cell death [2]. Parkinson’s disease, malignancies, Alzheimer’s disease, and diabetes are all linked to the overproduction of reactive oxygen species [3,4]. Redox balance between pro- and antioxidants is critical in treating and preventing many diseases. The use of antioxidants is generally restricted by their sensitivity to light, oxygen and pH, as well as their poor solubility in physiological fluid, low bioavailability and ineffective transport to undesirable cellular compartments, even if their potential is tremendous [5,6,7]. Researchers are always searching for new antioxidant species to promote healthy aging and prevent oxidative stress.

Nanoparticles can act as smart nanocarriers and have various applications including drug delivery. The combined effect of material science with nanotechnology and engineering has led to important developments that decrease free radicals’ production [8]. ‘Nanoantioxidants’ are smart nanocarriers with antioxidant capabilities that have been developed in recent years through the application of nanotechnology [9]. Using nanoantioxidant systems means that many of the limitations of standard antioxidant molecules could be overcome and their efficiency could be increased. Nanoantioxidant systems can exhibit prolonged stability and improved bioavailability. They have the capacity to evade quick metabolic activities, and the potential to give a regulated and targeted delivery [10]. The surface of smart nanocarriers can be functionalized with antioxidant molecules to transform nanoparticles into nanoantioxidants. In recent years, the surface functionalization of nanoparticles with antioxidants has been used to improve their biostability, biocompatibility and their ability to boost immune system [11]. Specifically, the simultaneous loading and functionalization of nanocarriers with antioxidants provides the advantage of delivering high amounts of antioxidants and the possibility for the co-delivery of other drugs and, thus, the use of these devices to exploit any synergic effects [12]. The surface functionalization of nanoparticles with natural antioxidants also imparts specific biological activity, which mainly depends upon the material used for functionalization, such as anticancer, antimicrobial, anti-Alzheimer’s and antidiabetic materials. Rutin and caffeic acid-functionalized silica nanoparticles were synthesized by Elle et al. and showed promising results, minimizing ROS production [13]. DPPH assay and radical scavenging assay of Gold nanoparticles (AuNP) immobalized on Kraft paper and cellulose fibre was performed in both dark and light conditions. [14]. Polyethylene glycol (C_2n_H_4n+2_O_n+1_), PEG-coated gold (AuNPs) was functionalized using the antioxidant of salvianic (C_9_H_10_O_5_) acid (Au@PEG3SA). The antioxidant properties of the functionalized Au@PEG3SA was observed. The free radical scavenging rate of Au@PEG3SA was nine times higher than that of the plain salvianic acid A monomers [15]. A new potent nano-antioxidant of sulfur-containing butylated hydroxytoluene ligands (S-BHTLs) conjugated with gold nanoparticles, Au-S-BHTLs, was synthesized by the conjugation of sulfur-containing ligands derived from BHT on the surface of gold nanoparticles (AuNPs). The in-house-developed eight sulfur-containing BHT-ligands (S-BHTLs) were used for further study on functionalization with AuNPs and their biological activities [16]. The antioxidant properties of iron oxide nanoparticles has already been studied and it has been shown that radical scavenging is due to electron transfer [17,18,19]. In another study, gallic acid and quercetin functionalized magnetite nanoparticles showed synergistic organic–inorganic hybrid antioxidant properties and potent antimicrobial activity on various fungal and bacterial strains [19,20].

Among the most commonly used synthetic antioxidants is butylated hydroxytoluene (BHT), with many reports confirming potent antioxidant activity in various industrial applications, such as in the food, oil, and cosmetics industries [21]. In addition, this synthetic phenolic antioxidant has also been applied in therapeutic fields; however, certain factors, such as volatility, high-temperature instability and toxicity and safety concerns, have greatly limited the effective therapeutic application of this antioxidant [22]. To this end, current research focuses on designing and synthesizing new BHT-derivatives to enhance antioxidant and therapeutic activities and reduce toxic side effects [5]. This study aimed to design and synthesize EG-ester of BHT bearing antioxidant groups as an effective strategy to enhance the safety profile, solubility of BHT, and synthesis of new multipotent antioxidant (MPAO) functionalized magnetic nanoantioxidant. Prior to the synthesis of the MPAO, computational studies were carried out to verify whether the designed molecules were based on a structure-activity relation (SAR) strategy. Rule of five, polar surface area and Lipinski parameters were used for predicting ADMET properties. PASS analysis was performed for the MPAO to predict the potential biological activities of the molecule. A post functionalization technique was used to synthesize magnetic nanoantioxidants. Antioxidant assay and antimicrobial activities were carried out for the functionalized nanoparticles IONP@AO.

## 2. Results

### 2.1. FTIR Analysis

**Figure 1:**
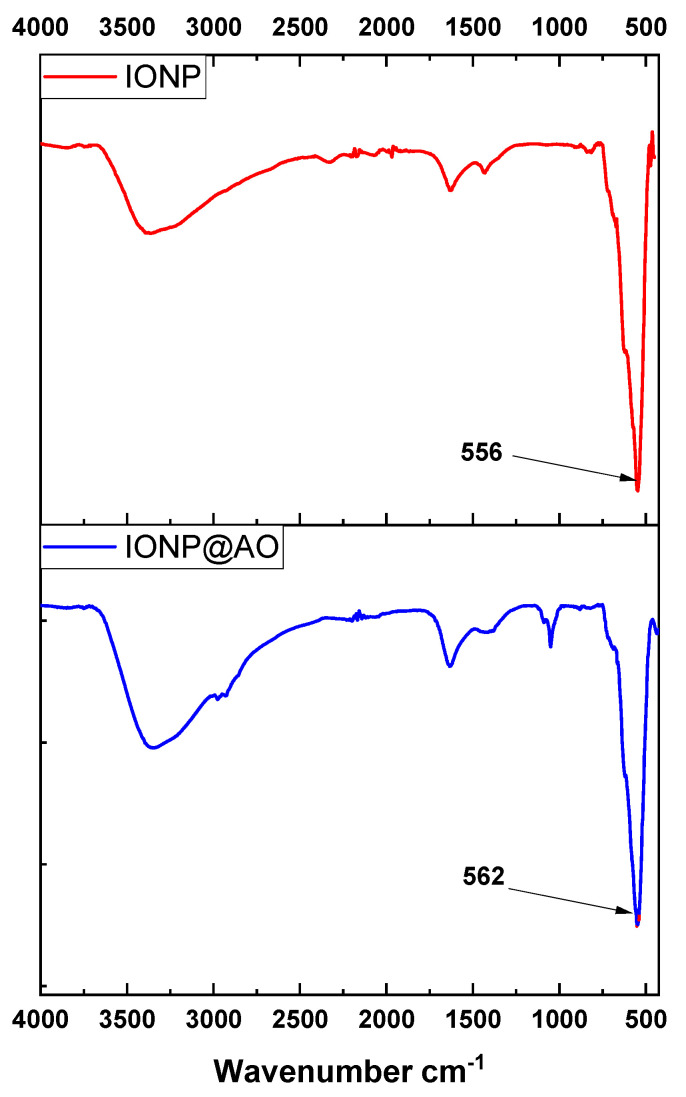
Surface Functional Groups identification using Fourier-transform infrared spectra of IONP@AO.

The FTIR spectra of iron oxide nanoparticles (IONP) and antioxidant functionalized iron oxide nanoparticles IONP@AO are shown in Figure 1 Magnetite was observed in the nanoparticle samples by a strong absorption at 556 and 562 cm^−1^ for IONP and IONP@AO, respectively, which corresponds to Fe-O stretching vibrations [23]. All the peaks represent the hydroxyl, carboxylic and aromatic groups present in organic molecules. The broad peak at 3100–3200 cm^−1^ represents the-OH stretching vibration. The peak at 1621 cm^−1^ confirms the existence of carbonyl groups in IONP@AO [24].

### 2.2. Raman Spectra

**Figure 2:**
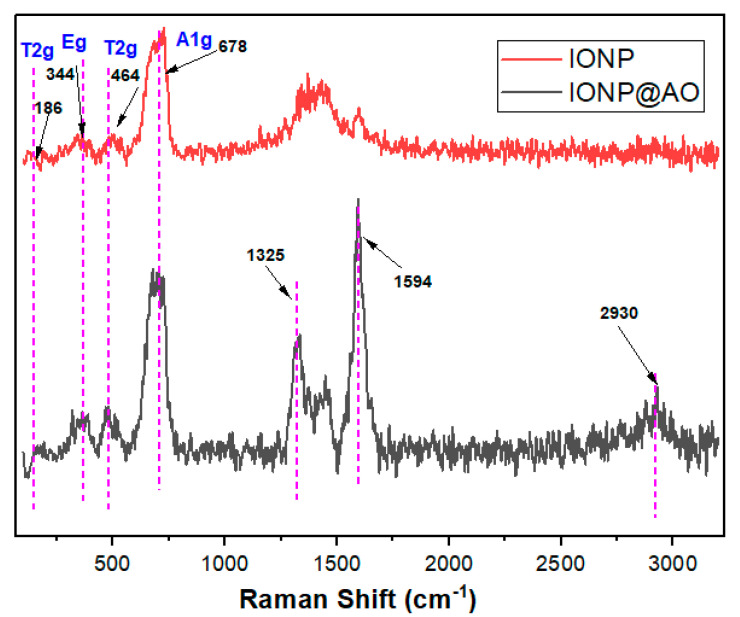
Raman spectra of IONP@AO.

Figure 2 shows Raman spectra of the functionalized IONP and unfunctionalized IONP. The main band confirms the presence of magnetite at 678 cm^−1^ (A1g) [11]. IONP@AO have a main band centred at 678 cm^−1^, and the peaks at ca. 464 cm^−1^ and 344 cm^−1^ are due to A1g, T2g and Eg vibrations of magnetite. The Raman spectra confirms that the samples did not contain maghemite [25,26].

### 2.3. XRD Analysis

**Figure 3:**
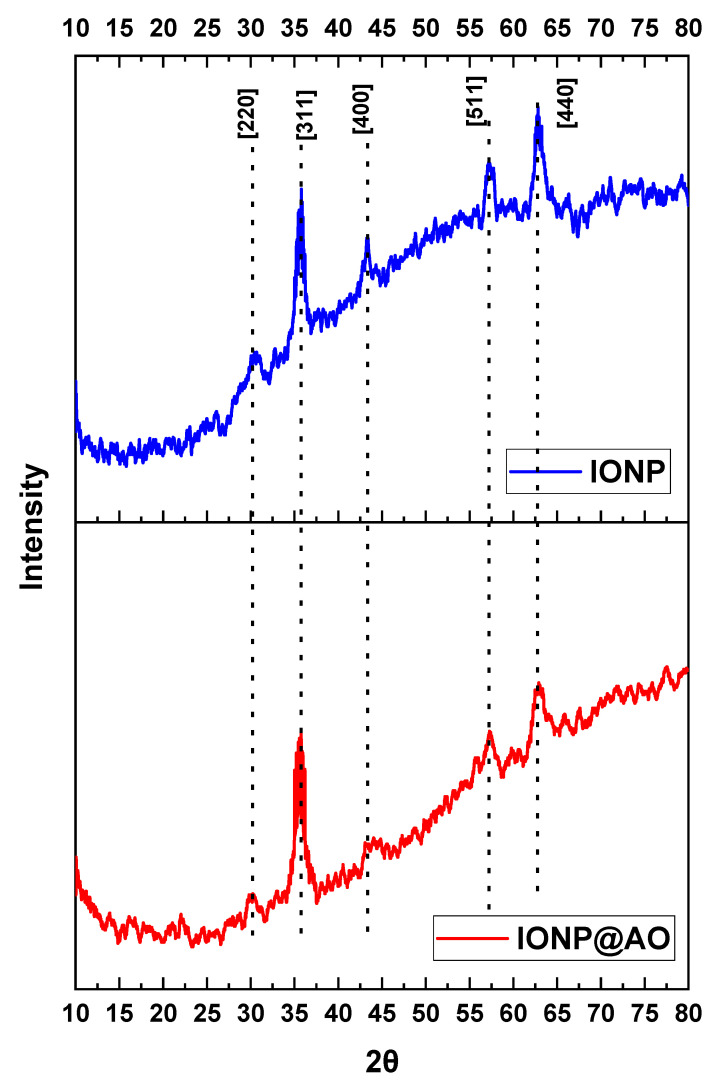
X-ray Diffraction spectra of IONP@AO.

XRD spectra for IONP and IONP@AO are shown in Figure 3. Diffraction peaks were observed in all samples at 2θ values of 30, 25, 43, 57, and 63, which correspond to Brag reflections in [220], [311], [400], [422] and [440] planes, respectively. The magnetite nanoparticles synthesized here have a cubic inverse spinal framework based on the XRD pattern (JCPDS No. 82-1533). The crystallinity index for functionalized and un-functionalized IONPs were 24.19 and 30.99%, respectively. The superlattice diffraction at 210, 213 and 300 were not present, confirming the absence of maghemite in the sample. Furthermore, no phase change was observed, which confirms that functionalization with organic moieties did not affect the magnetite phase.

### 2.4. Magnetic Properties

**Figure 4:**
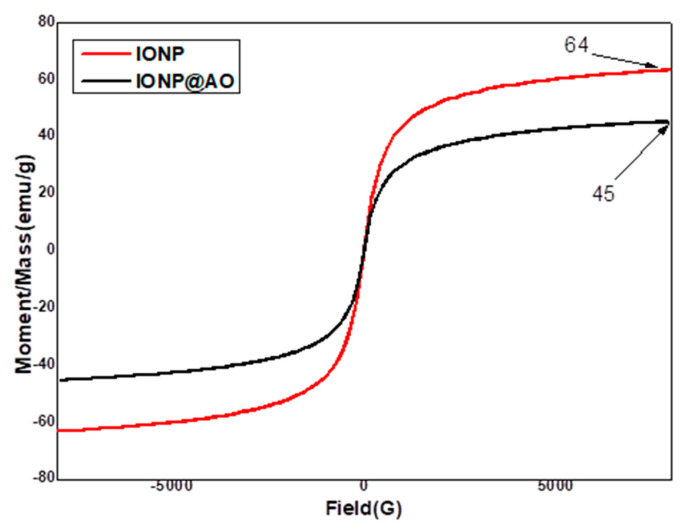
VSM of IONP@AO.

**Figure 5:**
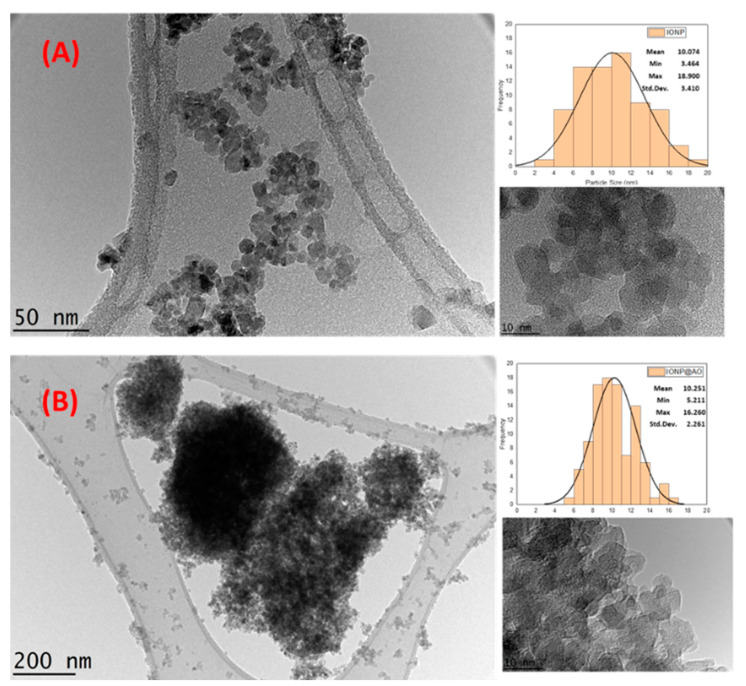
HRTEM images (**A**) Unfunctionalized IONP. (**B**) functionalized IONP@AO showing particle size distribution.

A Vibrating Sample Magnetometer (VSM) was used to determine saturated mass magnetization. The values of 64.19 and 45 emu g^−1^ were given for bare iron oxide nanoparticles and functionalized IONP@AO, respectively. Figure 4 shows the hysteresis loops as a function of the magnetic field at room temperature. The hysteresis loops are shown in Figure 5 as functions of the magnetic field at room temperature. All samples showed superparamagnetic behaviour, and their saturation magnetization was lower when compared to bulk Magnetite (92 emu g^−1^ [27]. The magnetization value for IONPs functionalized with *Camellia sinensis* L. showed a lower mag value 11 emu/g [28]. The decrease in saturation magnetization of IONP@AO over the surface of the produced nanoparticles is most likely due to organic molecules and impurities [29,30,31].

### 2.5. Morphological and Structural Studies

High-Resolution Transmission Electron Microscopy (HRTEM) was used to analyze the morphology of IONP@AO. HRTEM image and size distributions for IONP and IONP@AO are shown in Figure 5. The TEM images reveal that the mean particle size was 10.07 and 10 nm for IONP and IONP@AO, respectively. The particles are spherical in shape and have a homogeneous size distribution. Earlier research using by HRTEM analysis on functionalized IONPs with extract of *Camellia sinensis* L found spherical particles had the size of 20–35 nm [28]. The magnetic behavior of the samples causes the aggregation of iron oxide nanoparticles. The lattice fringe spacing of 0.26 corresponds to (220) lattice pane of magnetite nanoparticles [32].

### 2.6. EDX Analysis

**Figure 6:**
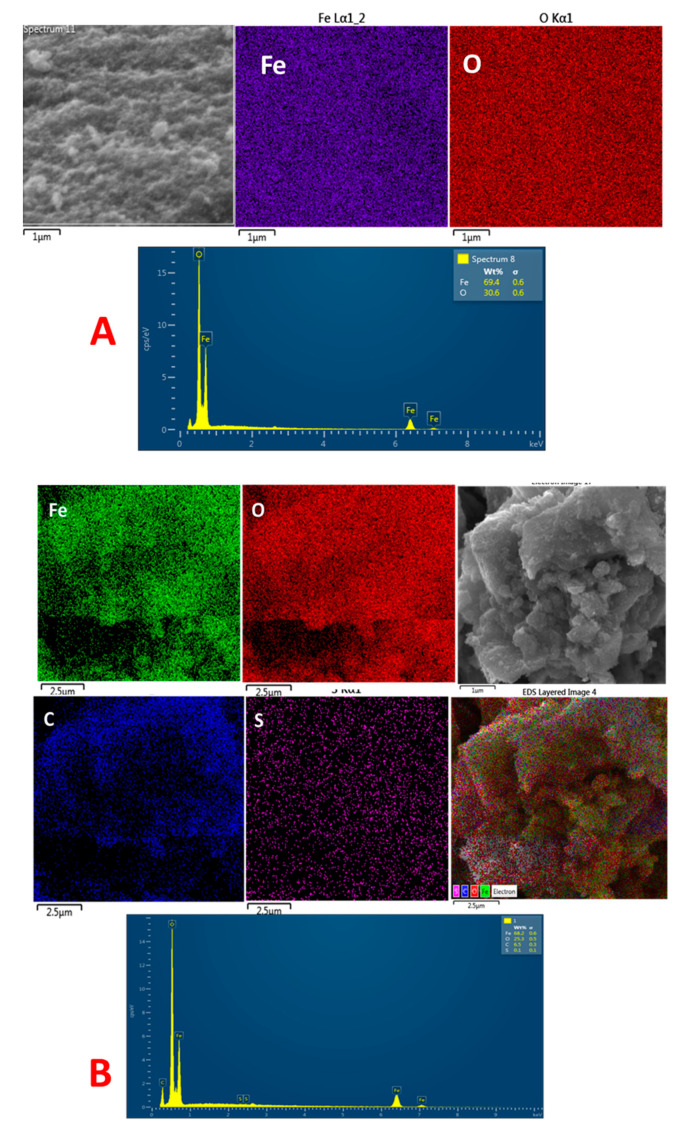
(**A**) FESEM image, EDX and elemental map of Fe, O of IONP. (**B**) FESEM image, EDX and elemental map of Fe, O, C and S of IONP@AO.

An energy dispersive X-ray spectroscopy (EDX) analysis was used to determine the elements in IONP and IONP@AO, respectively. Table 1 shows the elemental analysis of the synthesized IONP@AO. Figure 6 shows the EDX and elemental map of Fe, O, C and S for functionalized and unfunctionalized IONP. The EDX spectrum of the IONP@AO consisted of different peaks for Fe, O, C and S, confirming the successful formation of IONP@AO. The Fe and O signals are due to iron oxide, while carbon signals are due to an organic matrix. Furthermore, the IONP@AO elemental mapping revealed that the MPAO was spread uniformly throughout the microstructure of the IONP.

### 2.7. Computational Analysis

#### 2.7.1. ADMET Studies

**Figure 7:**
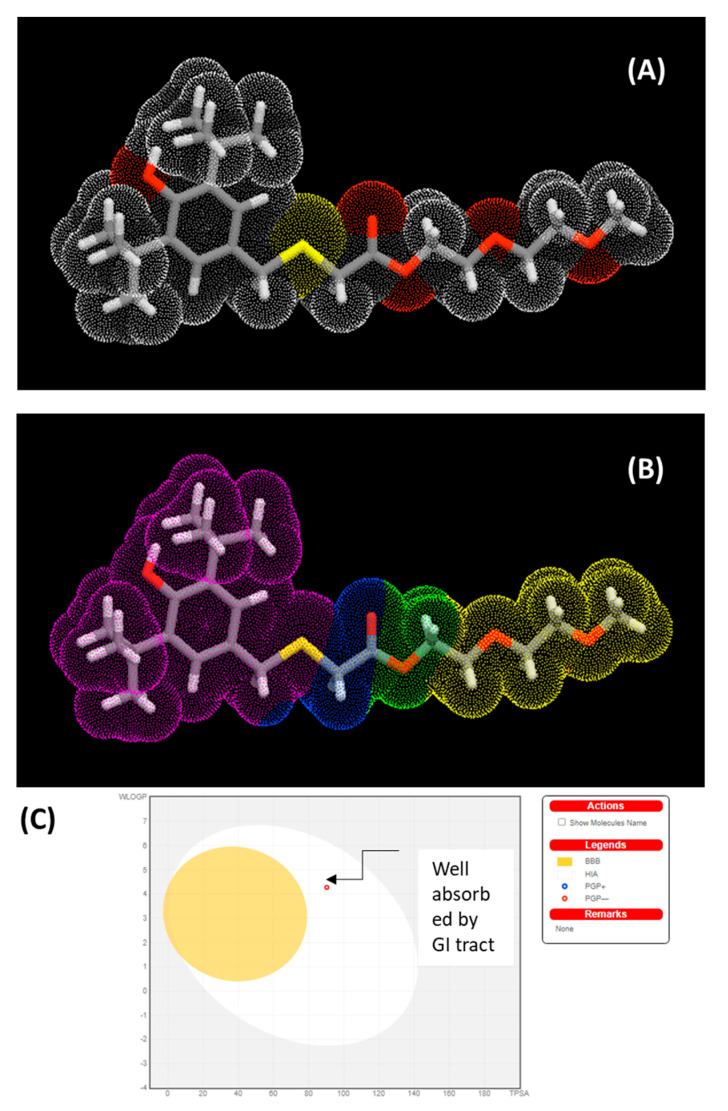
(**A**) polar surface area. (**B**) Molecular Lipophilicity Potential (MLP) and (**C**) boiled egg predictive model of MPAO.

The physicochemical characteristics of synthesized MPAO were analyzed and calculated based on Lipinski’s rule of five (Mol. Weight ≤ 500 Da, LogP ≤ 5, H-bond donor ≤ 5 and H bond accepter ≤ 10). Table 2 shows the properties predicted by ADMET. Figure 7A,B shows molecular lipophilicity potential (MLP) to visualize hydrophobicity (violet and blue colors) and hydrophilicity (orange and red) on the molecular surface. The miLogP method was used for MLP calculation from atomic hydrophobicity contributions; this method is the same as calculating the octanol-water partition coefficient (logP). MLP is valuable for rationalizing various molecular ADME characteristics (like membrane penetration or plasma-protein binding). 3D distribution of hydrophobicity on the molecule’s surface is helpful to explain the difference in observed ADME properties of molecules having the same logP values [33]. 3D parameters have more information than logP expressed by just a single value. Figure 7C shows the boiled egg predictive model of lipophilicity (WLOGP) and polarity (tPSA) computation. The white portion of the figure indicates a higher probability of absorption in the gastrointestinal system, whereas the yellow region (yolk) indicates a higher probability of brain permeation [34].

#### 2.7.2. PASS Analysis

The predicted bioactivities of synthesized compounds were predicted using the PASS model. Multi-level neighbour of atoms (MNA) descriptors (2D molecular fragment) are used in PASS studies, which show that biological activity is a function of molecular structure. The predictive score for activities is given as probability ratios between ‘probability to be active (Pa)’ and ‘probability of being non-active (Pi)’. Higher values of Pa represent the higher activity of organic molecules. Table 3 shows the selected bioactivities with higher Pa values when Pa > Pi (Table S1, Supplementary Material). Figure 7A,B shows the polar surface area and Molecular Lipophilicity Potential (MLP) of MPAO.

The fact that the MPAO has antioxidant values and other predicted bioactivities of Pa > 0.7 suggests that nanomaterial functionalized with the MPAO could display enhanced activities compared to nanoparticles without functionalization. This is owing to the MPAO’s biocompatibility, which can assist the drug transportation system as well as with bioimaging. Biological testing verified the predicted results.

### 2.8. Antioxidant Activity

**Figure 8:**
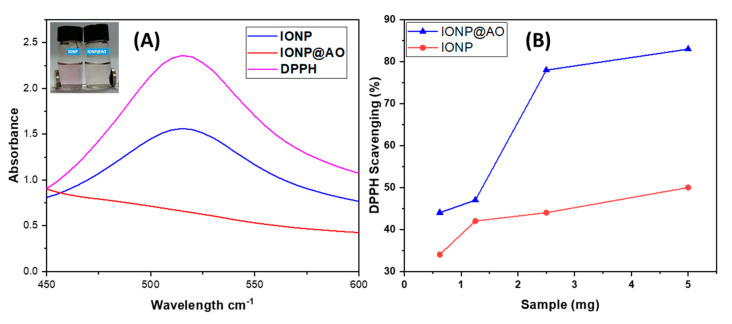
(**A**) UV–Visible Spectrum. (**B**) DPPH Scavenging percentage by IONP@AO at different concentrations.

Figure 8A shows UV–Visible spectra of the samples. The intensity of DPPH peak at 517 nm is decreasing. The IC50 value and the reduction in peak intensity were used to determine the free radical scavenging properties (Table 4).

The percent inhibition of stable free radical DPPH for synthesized nanoantioxidant was determined to be IONP@AO (1 ± 0.002 mg/mL; 83%) and at a 10^−4^ M, which is four times higher than unfunctionalized IONP (IC50 4.7 ± 0.002 mg/mL; 50%). In comparison to IONP, IONP@AO demonstrated greater free radical scavenging properties. Antioxidant activity depends on the amount of total antioxidant compounds present [35]. The nanoantioxidant scavenges free radicals by transferring electrons from functionalized IONP@AO to the center nitrogen atoms of the DPPH. The synergistic effect of IONP and the MPAO results in an increase in the free radical scavenging activity of IONP@AO. Similar results have been observed for Gallic acid and Quercetin functionalized IONPs [19,20]. Another study has reported that superparamagnetic iron oxide nanoparticles have good antioxidant activity because they are plant-extract-mediated (natural sweetener from stevia leaf extract) [36].

### 2.9. Antibacterial Activity

**Figure 9:**
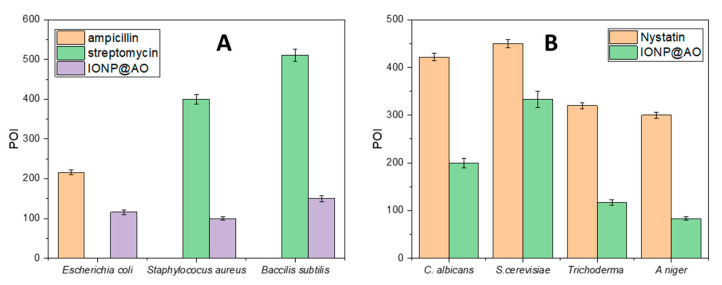
Percentage of inhibition (POI) of (**A**) bacterial growth and (**B**) fungal growth, after treatment with IONP@AO.

The results of the agar well diffusion technique are summarized in Figure 9A. The percentage inhibition of diameter growth (PIGD) of bacteria is plotted against the experimental sample concentration of 100 mg/mL. Antibacterial activity against Gram-negative and Gram-positive species of bacteria was observed for functionalized IONP@AO. For the most effective samples, the minimal inhibitory concentration was estimated. IONP@AO exhibited distinct bactericidal activity against Gram-positive and Gram-negative bacteria. Different varieties of bacteria had distinct types of cell wall, leading to this finding. Gram-positive bacteria have a relatively substantial, thicker peptidoglycan layer (10–30 nm) on their surface, while Gram-negative bacteria have an additional outer layer with a thin layer of peptidoglycan (10 nm). IONP@AO has been shown to have varying degrees of antibacterial activity against a range of bacterial species. When IONP@AO is added to bacterial strains, inhibition occurs due to the internalization of functionalized IONPs within the cells. This ultimately destroys the cell wall by breaking the 1,4 glycosidic linkages.

### 2.10. Antifungal Activity

The results obtained for an agar well diffusion method are illustrated in Figure 9B. Antifungal activity was observed for *Aspergillus Niger*, *Trichoderma* spp., *Candida albicans*, and *Saccharomyces cerevisiae*. In the cases of *Aspergillus Niger, Saccharomyces cerevisiae* and *Candida albicans*, IONP@AO showed enhanced antifungal activity. It exhibited reduced antifungal activity for *Trichoderma* sp. Functionalized nanoparticles eventually prompted the cellular damage and death of the treated cells. In general, ultra-small nanoparticles have fungicidal activities. This is determined by the nanoparticles’ synthesis protocol and physicochemical attributes.

## 3. Materials and Methods

### 3.1. Materials

IONPs were prepared using ferric chloride hexahydrate (FeCl_3_·6H_2_O, Sigma, Saint Louis, MO, USA, ≥97%), ferrous chloride tetrahydrate (FeCl_2_·4H_2_O, Merck, (Saint Louis, MO, USA), and ammonium hydroxide (R and M, 28%, Shanghai, China). All chemicals were of analytical grade and were used without further purification.

### 3.2. Chracterizations

The morphology of the functionalized nanoparticles was analyzed using a High-Resolution Transmission Electronic Microscope (HRTEM) (Model: JEM-2100F, JEOL, Tokyo, Japan). The system was equipped with a 200 kV field emission gun. To prepare samples for HRTEM a drop of the sample was evaporated on a carbon-coated copper grid. Gatan Digital MicroGraph software was used to measure particle size. Cu-Kα radiation (λ = 1.54060 Å) was utilized for XRD Analysis. The range of 2θ was scanned from 10.00 to 90.00 using a PANalytical X-ray diffractometer (Model: EMPYREAN, Almelo, The Netherlands). Surface functional groups were identified using Fourier-transform infrared spectroscopic analysis (Perkin Elmer, Boston, MA, USA. Energy dispersive X-ray analysis (EDX) (INCA Energy 200, Oxford Inst., Hillsboro, OR, USA) was performed under vacuum conditions and a working distance of 6 mm. Percentage composition was calculated using the surface area method. Raman spectra of the samples were taken on a Renishaw inVia Raman (Gloucestershire, UK) using a 514 nm Argon gas laser. Magnetic properties were measured in solid state at room temperature using VSM analysis (Lake Shore Magnetometer, Westerville, OH, USA). A LaboGene’s coolsafe freeze dryer was used for lyophilization.

### 3.3. Methods

#### 3.3.1. Computational Studies

A PASS web server was used to investigate potential biological activities of the MPAO. PASS is a useful tool for the exploration of possible bioactivities of organic molecules based on their chemical formula. Lipinski’s rule of five was applied to predict ADMET and physicochemical properties.

#### 3.3.2. Synthesis of MPAO

**Figure 10:**
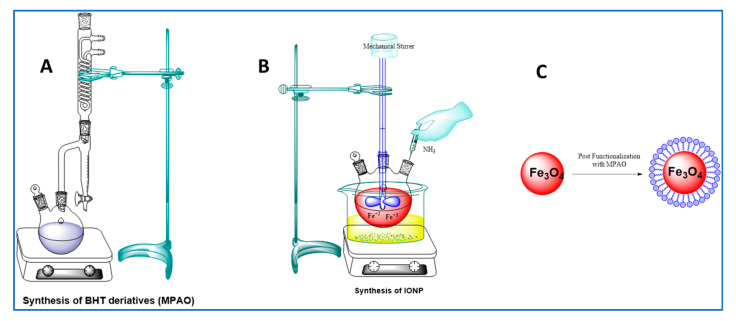
(**A**) Synthesis of MPAO. (**B**) Synthesis of IONP. (**C**) The functionalization of IONPs.

To a solution of 2-((3,5-di-tert-butyl-4-hydroxybenzyl)thio)acetic acid (2 g) in dry toluene (5 mL) was mixed with the respective MDEG. PTSA (0.02 g) was introduced into the above mixture, and the resultant solution was refluxed for 8 h. The water produced throughout the reaction was removed using the Dean–Stark system, as shown in Figure 10A. After cooling, the mixture was filtered and washed with distilled water to remove PTSA and unreacted EG and dried over anhydrous sodium sulphate. The precipitate was collected by filtration, dried at RT, and recrystallized from the appropriate solvent.

Molecular Formula: C_23_H_38_O_5_S, Molecular Weight: 426.61, ^1^H-NMR (600 MHz, CDCl_3_) δ 7.04, 5.09, 4.24, 4.23, 4.23, 3.71, 3.67, 3.66, 3.65, 3.59, 3.58, 3.57, 3.52, 3.51, 3.50, 3.46, 3.45, 3.44, 3.42, 3.07, 1.36, 1.13. ^13^C-NMR (151 MHz, CDCl_3_) δ 170.60, 153.01, 135.99, 127.53, 125.86, 70.69, 69.82, 69.02, 66.70, 36.76, 34.31, 32.56, 30.30, 15.11.

#### 3.3.3. Synthesis of IONP

An aqueous solution of FeCl_2_ and FeCl_3_ at a ratio of 1:1.5 (mole/mole) was prepared in 100 mL of DI water. 3 M NH_4_OH solution was mixed with the mixture of Fe salts at a rate of 5.0 mL min^−1^ and was subject to continuous stirring at 600 rpm until it had a final pH of 11. The reaction mixture was subject to mechanical stirring for 90 min at 80 °C, as shown Figure 10B. Magnetic decantation was used to isolate the black precipitate which formed. The precipitates were thoroughly washed with DI water and CH_3_CH_2_OH and were finally freeze dried. The process of lyophilization or freeze drying involves the removal of water from a product after it has been frozen in order to allow it to transform directly from a solid state into a gaseous state [37]. The sample was frozen to a temperature below its “eutectic point” at −60 °C and then freeze dried at an ultra-low pressure.

#### 3.3.4. Functionalization

##### Synthesis of IONP@AO

The synthesized MPAO was dissolved in ethanol and was added to the ethanolic suspension of IONPs. The reaction mixture was sonicated for 20 min and after that it was stirred for 24 h. Deionized water and ethanol (C_2_H_5_OH) were used to thoroughly rinse the precipitates prior to freeze-drying. A schematic representation of IONP functionalization is shown in Figure 10C.

#### 3.3.5. Antioxidant Activity

Antioxidant activity can be evaluated by using various chemical-based methods.

Depending on the reaction, these assays can be divided into two categories: H atom transfer and electron transfer. The antioxidant activity of synthesized IONP@AO was investigated using the DPPH assays based on the electron transfer mechanism [38]. A modified DPPH assay was used to study antioxidant nature of nanoparticles [10,38]. 1 cm quartz cuvettes were used to prepare the mixture of 1 mL of the methanolic solution of DPPH (0.2 mM) and the methanolic suspension of the sample (300 μL). For each experiment, absorbance was recorded after thirty minutes. The readings were constantly taken at 517 nm. All readings were taken twice within thirty minutes after adding DPPH solution to the sample. The radical scavenging capacity was estimated by following Equation (1):

(1) “Inhibition Percentages” (%)=(Ac−As)/Ac×100        

where *As* = positive control/absorbance of the compound *Ac* = the absorbance of DPPH solution (control). At different concentrations, the percent inhibition obtained was different, and it was plotted to calculate IC_50_.

#### 3.3.6. Antimicrobial Activity

##### Determination of Antibacterial Activity

The agar well diffusion method was used to estimate the antibacterial activity of IONP@AO [39]. Bacterial precultures of *Bacillus sbustilis*, *Staphylococcus aureus* and *Escherichia coli* were spread over the Nutrient Agar surface, and 100 microliters of test samples (100 mg/mL) was added to the wells (6 mm diameter). The petri dishes were incubated for 24 h at 37 °C. Sterile DI water was used as a negative control, while ampicillin (100 mg/mL) and streptomycin 100 mg/disc were utilized as a positive control for Gram-negative and Gram-positive bacterial strains, correspondingly. The antibacterial properties were evaluated by measuring halo (inhibition) zones.

##### Determination of Antifungal Activity

To determine antifungal properties, functionalized and unfunctionalized IONPs were tested against various fungal strains by using the agar well diffusion method. *Aspergillus Niger*, a filamentous fungus (multicellular), *Saccharomyces cerevisiae*, a yeast (unicellular) and *Candida albicans*, a yeast and *Trichoderma* spp. were used in this work. Fungal strains were inoculated with potato dextrose agar (PDA) plates under aspectic conditions.

The wells were filled with 100 L of the test sample (100 mg/mL) and incubated at 25 °C for 48 h.

Incubation was done at 25 °C for 48 h in 100 L of the test sample (100 mg/mL).

As a negative control, sterile DI water was utilized. Sterile distilled water and Nystatin (100 mg/mL) was used as negative and positive controls respectively. The *POI*, representing the growth of mycelia, was estimated using Equation (2):

(2) $$POI=\frac{R1-R2}{R1}\times 100$$

where *R*1 = the radius of the pathogen away from the antagonist and *R*2 = the radius of the pathogen towards the antagonist.

## 4. Conclusions

MPAO functionalized IONP was successfully synthesized using a post-functionalization procedure. For post-functionalized IONP@AO, the average particle size was 10 nm. IC50 for IONPs were 4.7 ± 0.002. However, the functionalized IONPs@AO showed IC50 values 1 ± 0.002 mg/mL. The IONP@AO was studied using XRD, FTIR, VSM, EDX, HRTEM and Raman analysis, which demonstrated that it had properties similar to magnetite. The superparamagnetic nature of the produced nanoparticles was confirmed by VSM. In order to uncover and anticipate the molecule’s potential bioactivities and safety profile, the structure-based virtual screening of the MPAO was carried out using PASS analysis and ADMET studies. IONP@AO showed better radical scavenging and antimicrobial activities. The MPAO functionalized IONP showed promising free radical scavenging.

## Figures and Tables

**Table 1:** EDX Elemental Analysis of IONP@AO.

Sample	Fe	O	C	S
IONP	69.4	30.6	-	-
IONP@AO	68.2	25.3	6.5	0.1

**Table 2:** Predicted ADMET Properties from Computational Analysis.

**Physicochemical Properties**	
Number of rotatable bonds	13
Number of H-bond acceptors	5
Number of H-bond donors	1
MR	116.67
TPSA	90.29
**Lipophilicity**	
iLOGP	4.58
XLOGP3	4.98
WLOGP	4.27
MLOGP	2.99
Silicos-IT LogP	5.62
Consensus LogP	4.49
**Water Solubility**	
ESOL Log S	−4.84
ESOL Solubility (mg/mL)	6.02 × 10^−3^
ESOL Solubility (mol/l)	1.46 × 10^−5^
ESOL Class	Moderately soluble
**Pharmacokinetics**	
GI absorption	High
BBB permeant	No
Pgp substrate	No
CYP1A2 inhibitor	No
CYP2C19 inhibitor	No
CYP2C9 inhibitor	No
CYP2D6 inhibitor	Yes
CYP3A4 inhibitor	Yes
log Kp (cm/s)	−5.28
**Druglikeness**	
Lipinski number of violations	0
Ghose number of violations	0
Veber number of violations	1
Egan number of violations	0
Muegge number of violations	0
Bioavailability Score	0.55
**Medicinal Chemistry**	
PAINS number of alerts	0
Brenk number of alerts	0
Leadlikeness number of violations	3
Synthetic Accessibility	3.89

**Table 3:** Part of the predicted biological activity spectra of the MPAO based on PASS prediction software.

^a^ Pa	^b^ Pi	Biological Activity
0.456	0.013	Free radical scavenger
0.351	0.049	Lipid peroxidase inhibitor
0.285	0.026	Antioxidant
0.268	0.097	Antifungal
0.224	0.098	Antibacterial

^a^ Probability “to be active”. ^b^ Probability “to be inactive”.

**Table 4:** IC50 of IONP@AO.

IC50 ^a^ Values (mg) ± S.E.M ^b^ and Max. Inhibition %			
Sample		IC50 mg/mL	% Inhibition
IONP	5 mg	4.7 ± 0.002	50
IONP@AO	5 mg	1 ± 0.002	83

^a^ IC50, 50% effective concentration. ^b^ S.E.M, standard error of the mean.

## Data Availability

Not applicable.

## Supplementary Materials

The following are available online, Table S1: Part of the Predicted Biological Activity Spectra of the MPAO based on PASS Prediction Software.

## Sample Availability

Samples of the compounds are not available from the authors.

## Abbreviations

ADME	Absorption, Distribution, Metabolism and Excretion.
ADMET	Absorption, Distribution, Metabolism, Excretion and Toxicity
AO	Antioxidant
BBB	Blood–Brain Barrier
BHT	Butylated Hydroxytoluene
DI	Deionized Water
DNA	Deoxyribonucleic Acid
DPPH	2,2-diphenyl-1-picrylhydrazyl
EDX	Energy Dispersive X-ray Analysis
FESEM	Field Emission Scanning Electron Microscope
FTIR	Fourier-transform infrared spectroscopy
HRTEM	High-Resolution Transmission Electron Microscopy
IC_50_	Half-maximal inhibitory concentration
IONP	Iron Oxide Nanoparticle
MLP	Molecular Lipophilicity Potential
MNA	Multi-level Neighbour of Atoms
MPAO	Multipotent Anioxidant
NMR	Nuclear Magnetic Resonance Spectroscopy
NP	Nanoparticles
PASS	Prediction of Activity Spectra for Biologically Active Substances
PDA	Potato Dextrose Aagar
PEG	Polyethylene Glycol
PIGD	Percentage Inhibition of Diameter Growth
POI	Percentage of Inhibition
PSA	Polar Surface Area
PTSA	*p*-Toluenesulfonic acid
ROS	Reactive Oxygen Species
SAR	Structure Activity Relation
TEM	Transmission Electron Microscopy
TPSA	TotalPolar Surface Area
UV	Ultraviolet
VSM	Vibrating-Sample Magnetometer
XRD	X-ray Crystallography
