# Supplementary material for: Surface Functionalization of Magnetite Nanoparticles with Multipotent Antioxidant as Potential Magnetic Nanoantioxidants and Antimicrobial Agents
Source: Molecules. 2022 Jan 25;27(3):789. doi: 10.3390/molecules27030789 (PMC8840749; doi:10.3390/molecules27030789)
Supplement: Supplementary file 1 [file molecules-27-00789-s001.zip › molecules-1335521-supplementary.pdf]

molecules

MDPI

Supplementary Material

Surface Functionalization of Magnetite Nanoparticles with Multipotent Antioxidant as Potential Magnetic Nanoantioxidants and Antimicrobial Agents

Syed Tawab Shah [1], Zaira Zaman Chowdhury [1,*], Mohd. Rafie Bin Johan [1], Irfan Anjum Badruddin [2,3], H. M. T. Khaleed [4], Sarfaraz Kamangar [2] and Hussein Alrobei [5]

1 Nanotechnology and Catalysis Research Center, University of Malaya, Kuala Lumpur 50603, Malaysia; tawab_shah2003@yahoo.com (S.T.S.); mrafiej@um.edu.my (M.R.B.J.)
2 Department of Mechanical Engineering, College of Engineering, King Khalid University, Abha 61421, Saudi Arabia; irfan@kku.edu.sa (I.A.B.); shiblygsa@gmail.com (S.K.)
3 Research Center for Advanced Materials Science (RCAMS), King Khalid University, Abha 61413, Saudi Arabia
4 Department of Mechanical Engineering, Faculty of Engineering, Islamic University of Madinah, Medina 42351, Saudi Arabia; khalid_tan@yahoo.com
5 Department of Mechanical Engineering, Faculty of Engineering, Prince Sattam Bin Abdulaziz University, Al-Kharj 16278, Saudi Arabia; h.alrobei@psau.edu.sa
* Correspondence: dr.zaira.chowdhury@um.edu.my or zaira.chowdhury76@gmail.com; Tel: +60-37-967-2929 or +60-10-267-5621

Table S1. Part of the Predicted Biological Activity Spectra of the MPAO based on PASS Prediction Software.

Pa	Pi	Biological Activity
**0.884**	0.004	Skin irritation, inactive
**0.861**	0.008	Antiseborrheic
**0.811**	0.016	Mucomembranous protector
**0.778**	0.006	Lipoprotein lipase inhibitor
**0.738**	0.008	Radioprotector
**0.727**	0.004	Acaricide
**0.726**	0.005	Eye irritation, inactive
**0.678**	0.016	Hypolipemic
**0.671**	0.017	Fucosterol-epoxide lyase inhibitor
**0.651**	0.013	Cholesterol antagonist
**0.620**	0.019	Lipid metabolism regulator
**0.605**	0.019	Linoleate diol synthase inhibitor
**0.624**	0.042	Alkenylglycerophosphocholine hydrolase inhibitor
**0.602**	0.024	General pump inhibitor
**0.652**	0.079	Aspulvinone dimethylallyltransferase inhibitor
**0.583**	0.014	Hepatoprotectant
**0.595**	0.033	Antiinflammatory
**0.573**	0.013	Antiulcerative
**0.571**	0.014	Anesthetic general
**0.568**	0.036	Feruloyl esterase inhibitor

**0.550**	0.020	TNF expression inhibitor
**0.538**	0.014	Acetylgalactosaminyl-O-glycosyl-glycoprotein beta-1,3-N-acetylglucosaminyltransferase inhibitor
**0.538**	0.041	Phosphatidylcholine-retinol O-acyltransferase inhibitor
**0.509**	0.015	Antiparasitic
**0.537**	0.045	Venombin AB inhibitor
**0.542**	0.051	Cytoprotectant
**0.545**	0.058	Acute neurologic disorders treatment
**0.507**	0.025	1-Alkylglycerophosphocholine O-acetyltransferase inhibitor
**0.503**	0.024	Reductant
**0.586**	0.113	Ubiquinol-cytochrome-c reductase inhibitor
**0.499**	0.035	Kinase inhibitor
**0.542**	0.084	Antiischemic, cerebral
**0.504**	0.050	Lysine 2,3-aminomutase inhibitor
**0.477**	0.025	Antihypercholesterolemic
**0.456**	0.013	Free radical scavenger
**0.449**	0.006	ICAM1 expression inhibitor
**0.489**	0.047	Membrane integrity antagonist
**0.474**	0.033	Antiallergic
**0.481**	0.047	Antianginal
**0.462**	0.038	Antisecretoric
**0.458**	0.042	Alkenylglycerophosphoethanolamine hydrolase inhibitor
**0.469**	0.057	Leukopoiesis stimulant
**0.414**	0.005	Retinoprotector
**0.436**	0.033	Antimyopathies
**0.417**	0.016	Ethanolaminephosphotransferase inhibitor
**0.402**	0.009	Antidote
**0.430**	0.043	All-trans-retinyl-palmitate hydrolase inhibitor
**0.470**	0.083	Protein-disulfide reductase (glutathione) inhibitor
**0.447**	0.063	Thromboxane B2 antagonist
**0.412**	0.035	Atherosclerosis treatment
**0.402**	0.027	Vanilloid 1 agonist
**0.372**	0.014	Anticataract
**0.378**	0.021	Ubiquitin thiolesterase inhibitor
**0.366**	0.013	ErbB-1 antagonist
**0.394**	0.043	1-Acylglycerol-3-phosphate O-acyltransferase inhibitor
**0.354**	0.002	Glycine receptor agonist
**0.364**	0.013	Expectorant
**0.375**	0.028	Ophthalmic drug
**0.444**	0.100	Proteasome ATPase inhibitor
**0.352**	0.010	Restenosis treatment
**0.353**	0.014	Anti-Helicobacter pylori
**0.428**	0.091	NADPH peroxidase inhibitor

**0.347**	0.012	Antiprotozoal (Trichomonas)
**0.428**	0.095	Mannotetraose 2-alpha-N-acetylglucosaminyltransferase inhibitor
**0.401**	0.070	Alcohol O-acetyltransferase inhibitor
**0.406**	0.076	Trimethylamine-oxide aldolase inhibitor
**0.364**	0.046	Phosphatidylinositol diacylglycerol-lyase inhibitor
**0.320**	0.006	Lipoxygenase inhibitor
**0.315**	0.003	Antidote, heavy metal
**0.401**	0.090	Histamine release inhibitor
**0.401**	0.091	Alkylacetylglycerophosphatase inhibitor
**0.344**	0.041	Carboxypeptidase D inhibitor
**0.351**	0.049	Lipid peroxidase inhibitor
**0.297**	0.007	Phospholipase A2 inhibitor
**0.374**	0.086	Ecdysone 20-monooxygenase inhibitor
**0.312**	0.023	Retinol dehydrogenase inhibitor
**0.331**	0.044	Antinephritic
**0.303**	0.016	Poly(3-hydroxybutyrate) depolymerase inhibitor
**0.330**	0.043	Antiinflammatory, ophthalmic
**0.395**	0.114	Glyceryl-ether monooxygenase inhibitor
**0.365**	0.086	Pancreatic elastase inhibitor
**0.329**	0.050	Acidifying agent non gastric
**0.282**	0.004	VCAM1 expression inhibitor
**0.310**	0.034	Antipruritic, non-allergic
**0.291**	0.015	Gastric antisecretory
**0.288**	0.015	Protein-S-isoprenylcysteine O-methyltransferase inhibitor
**0.303**	0.030	Alpha-amylase inhibitor
**0.286**	0.015	Mucolytic
**0.384**	0.116	Chlordecone reductase inhibitor
**0.317**	0.052	Anticarcinogenic
**0.366**	0.101	Apyrase inhibitor
**0.278**	0.016	Endoglycosylceramidase inhibitor
**0.285**	0.026	Antioxidant
**0.327**	0.069	Antidiabetic
**0.409**	0.152	Fusarinine-C ornithinesterase inhibitor
**0.273**	0.018	Cystinyl aminopeptidase inhibitor
**0.320**	0.067	Nitrite reductase (NO-forming) inhibitor
**0.327**	0.074	Mediator release inhibitor
**0.410**	0.158	Chymosin inhibitor
**0.410**	0.158	Acrocylindropepsin inhibitor
**0.410**	0.158	Saccharopepsin inhibitor
**0.383**	0.132	Sugar-phosphatase inhibitor
**0.289**	0.038	Chemoprotective
**0.273**	0.025	Lipoprotein disorders treatment

**0.365**	0.119	1,4-Lactonase inhibitor
**0.265**	0.020	Leukotriene synthesis inhibitor
**0.284**	0.039	Antiseptic
**0.297**	0.052	Levansucrase inhibitor
**0.253**	0.013	Stromelysin 2 inhibitor
**0.287**	0.047	Non-steroidal antiinflammatory agent
**0.329**	0.090	Phenol O-methyltransferase inhibitor
**0.318**	0.079	Dermatologic
**0.322**	0.083	Retinoic acid metabolism inhibitor
**0.307**	0.069	FMO3 substrate
**0.384**	0.150	TP53 expression enhancer
**0.267**	0.034	Protein-tyrosine sulfotransferase inhibitor
**0.335**	0.102	Peptide agonist
**0.274**	0.042	Choleretic
**0.289**	0.058	Maillard reaction inhibitor
**0.241**	0.011	Antiemphysemic
**0.333**	0.103	Caspase 8 stimulant
**0.324**	0.094	N-acetylneuraminate 7-O(or 9-O)-acetyltransferase inhibitor
**0.233**	0.003	Prostaglandin EP4 antagonist
**0.279**	0.050	Pediculicide
**0.241**	0.015	Phospholipase inhibitor
**0.301**	0.076	Antipsoriatic
**0.284**	0.059	Methane monooxygenase inhibitor
**0.346**	0.121	Acylcarnitine hydrolase inhibitor
**0.307**	0.085	Antiasthmatic
**0.261**	0.041	Acyl-CoA oxidase inhibitor
**0.282**	0.062	Photosensitizer
**0.278**	0.061	CYP2E1 substrate
**0.235**	0.019	Arachidonic acid antagonist
**0.253**	0.038	Histone acetyltransferase inhibitor
**0.252**	0.037	Antinaupathic
**0.262**	0.049	Anesthetic
**0.346**	0.133	Preneoplastic conditions treatment
**0.240**	0.028	Diacylglycerol cholinephosphotransferase inhibitor
**0.318**	0.108	Superoxide dismutase inhibitor
**0.291**	0.082	Neuropeptide Y2 antagonist
**0.236**	0.026	Retinyl-palmitate esterase inhibitor
**0.267**	0.058	Ferredoxin hydrogenase inhibitor
**0.237**	0.028	Microtubule formation inhibitor
**0.230**	0.022	Methylglutamate dehydrogenase inhibitor
**0.215**	0.007	5-Lipoxygenase inhibitor
**0.284**	0.078	4-Coumarate-CoA ligase inhibitor

**0.268**	0.061	Hepatic disorders treatment
**0.232**	0.026	Calcium channel blocker
**0.270**	0.064	CYP2E substrate
**0.424**	0.219	Phobic disorders treatment
**0.308**	0.104	Peptidoglycan glycosyltransferase inhibitor
**0.339**	0.135	Aspartyltransferase inhibitor
**0.272**	0.069	Arylesterase inhibitor
**0.205**	0.003	Prostaglandin E2 agonist
**0.337**	0.141	Oxygen scavenger
**0.346**	0.150	NADPH-cytochrome-c2 reductase inhibitor
**0.264**	0.068	Clavaminate synthase inhibitor
**0.227**	0.034	Aldehyde dehydrogenase inhibitor
**0.215**	0.023	O-acetylhomoserine aminocarboxypropyltransferase inhibitor
**0.361**	0.170	Glycosylphosphatidylinositol phospholipase D inhibitor
**0.261**	0.071	Undecaprenyl-phosphate mannosyltransferase inhibitor
**0.299**	0.110	Antihelmintic (Nematodes)
**0.265**	0.077	Dextranase inhibitor
**0.259**	0.072	Phosphatidylcholine-sterol O-acyltransferase inhibitor
**0.333**	0.146	Glucan endo-1,3-beta-D-glucosidase inhibitor
**0.309**	0.122	Apoptosis agonist
**0.247**	0.061	Shikimate O-hydroxycinnamoyltransferase inhibitor
**0.206**	0.022	Leishmanolysin inhibitor
**0.210**	0.026	Skin whitener
**0.310**	0.126	GST A substrate
**0.217**	0.035	Antiglaucomic
**0.232**	0.050	Vanilloid agonist
**0.230**	0.048	Inorganic diphosphatase inhibitor
**0.261**	0.080	Antitoxic
**0.274**	0.094	Methylenetetrahydrofolate reductase (NADPH) inhibitor
**0.291**	0.112	CYP3A4 inducer
**0.275**	0.096	CYP2C19 substrate
**0.235**	0.056	Glycine dehydrogenase (decarboxylating) inhibitor
**0.311**	0.132	5 Hydroxytryptamine release inhibitor
**0.241**	0.063	Cutinase inhibitor
**0.285**	0.108	Mucinaminylserine mucinaminidase inhibitor
**0.222**	0.044	Aldehyde dehydrogenase (NADP+) inhibitor
**0.296**	0.120	Antipruritic
**0.383**	0.207	Phosphatase inhibitor
**0.240**	0.065	Sclerosant
**0.307**	0.133	Hydrogen dehydrogenase inhibitor
**0.221**	0.048	Penicillin amidase inhibitor
**0.279**	0.106	HMOX1 expression enhancer

**0.289**	0.117	CDP-diacylglycerol-serine O-phosphatidyltransferase inhibitor
**0.276**	0.105	Phosphoinositide 5-phosphatase inhibitor
**0.268**	0.097	Antifungal
**0.276**	0.106	Plastoquinol-plastocyanin reductase inhibitor
**0.205**	0.035	Pectate lyase inhibitor
**0.259**	0.091	Pyruvate decarboxylase inhibitor
**0.190**	0.022	Adenylate cyclase stimulant
**0.240**	0.073	Lactose synthase inhibitor
**0.192**	0.025	Lactoylglutathione lyase inhibitor
**0.380**	0.213	Antieczematic
**0.235**	0.069	Di-trans,poly-cis-decaprenylcistransferase inhibitor
**0.277**	0.113	CYP3A inducer
**0.261**	0.096	Steroid N-acetylglucosaminyltransferase inhibitor
**0.337**	0.172	Electron-transferring-flavoprotein dehydrogenase inhibitor
**0.335**	0.172	Membrane integrity agonist
**0.270**	0.108	Cardioprotectant
**0.271**	0.110	3-Cyanoalanine hydratase inhibitor
**0.199**	0.038	Insecticide
**0.284**	0.123	CYP2A4 substrate
**0.290**	0.130	N-hydroxyarylamine O-acetyltransferase inhibitor
**0.340**	0.181	CYP2C8 inhibitor
**0.258**	0.099	Mannose isomerase inhibitor
**0.237**	0.078	Prolyl aminopeptidase inhibitor
**0.164**	0.006	Leukotriene antagonist
**0.221**	0.063	Cyclopropane-fatty-acyl-phospholipid synthase inhibitor
**0.284**	0.127	Transcription factor NF kappa B stimulant
**0.284**	0.127	Transcription factor stimulant
**0.194**	0.038	Melanin inhibitor
**0.188**	0.033	Carboxylesterase inhibitor
**0.229**	0.074	Thiol protease inhibitor
**0.299**	0.144	Antipruritic, allergic
**0.317**	0.162	APOA1 expression enhancer
**0.343**	0.189	5-O-(4-coumaroyl)-D-quinate 3'-monooxygenase inhibitor
**0.157**	0.005	Adenine nucleotide translocase inhibitor
**0.180**	0.029	Agmatinase inhibitor
**0.189**	0.039	Antihyperlipoproteinemic
**0.191**	0.041	Pyroglutamyl-peptidase I inhibitor
**0.228**	0.080	Dactylysin inhibitor
**0.245**	0.097	Pullulanase inhibitor
**0.279**	0.132	Sphinganine kinase inhibitor
**0.212**	0.065	Lipoprotein lipase stimulant
**0.180**	0.033	Alcohol dehydrogenase substrate

**0.278**	0.132	Leukotriene-C4 synthase inhibitor
**0.352**	0.206	Kidney function stimulant
**0.176**	0.031	Thiol S-methyltransferase inhibitor
**0.180**	0.035	Aspartyl aminopeptidase inhibitor
**0.213**	0.070	Antiuremic
**0.243**	0.100	Monodehydroascorbate reductase (NADH) inhibitor
**0.145**	0.003	Free fatty acid receptor 1 agonist
**0.285**	0.144	Benzoate-CoA ligase inhibitor
**0.272**	0.130	Manganese peroxidase inhibitor
**0.289**	0.148	Fragilysin inhibitor
**0.204**	0.064	Coenzyme-B sulfoethylthiotransferase inhibitor
**0.268**	0.127	Bilirubin oxidase inhibitor
**0.221**	0.082	UGT1A6 substrate
**0.329**	0.191	Ribulose-phosphate 3-epimerase inhibitor
**0.215**	0.078	CDP-diacylglycerol-glycerol-3-phosphate 3-phosphatidyltransferase inhibitor
**0.196**	0.060	Sterol 3-beta-glucosyltransferase inhibitor
**0.232**	0.097	Gamma-glutamyltransferase inhibitor
**0.185**	0.052	Cytosol alanyl aminopeptidase inhibitor
**0.278**	0.145	S-formylglutathione hydrolase inhibitor
**0.242**	0.110	Immunomodulator
**0.205**	0.073	Endopeptidase inhibitor
**0.162**	0.032	Caspase 3 inhibitor
**0.189**	0.059	Deoxyribonuclease I inhibitor
**0.219**	0.090	[acyl-carrier-protein] S-acetyltransferase inhibitor
**0.163**	0.034	Cyclooxygenase 1 inhibitor
**0.197**	0.068	Lymphocytopoiesis inhibitor
**0.241**	0.112	RNA synthesis inhibitor
**0.175**	0.047	Phosphoinositide phospholipase C inhibitor
**0.132**	0.004	Prostaglandin EP1 antagonist
**0.153**	0.025	Homocysteine S-methyltransferase inhibitor
**0.230**	0.103	Aspergillus nuclease S1 inhibitor
**0.192**	0.066	Anthrax lethal factor inhibitor
**0.224**	0.098	Antibacterial
**0.197**	0.071	Proteasome endopeptidase complex inhibitor
**0.341**	0.218	Nicotinic alpha4beta4 receptor agonist
**0.233**	0.111	UGT1A9 substrate
**0.127**	0.004	Prostaglandin EP2 antagonist
**0.186**	0.064	Isopenicillin-N synthase inhibitor
**0.227**	0.106	2-Hydroxymuconate-semialdehyde hydrolase inhibitor
**0.235**	0.115	N-formylmethionyl-peptidase inhibitor
**0.160**	0.040	Sarcosine oxidase inhibitor
**0.204**	0.085	Fumarate reductase (NADH) inhibitor

**0.205**	0.086	Pappalysin-1 inhibitor
**0.177**	0.059	Keratolytic
**0.247**	0.129	Dolichyl-diphosphooligosaccharide-protein glycotransferase inhibitor
**0.270**	0.154	Arginine 2-monooxygenase inhibitor
**0.370**	0.255	CYP2H substrate
**0.244**	0.129	Aldehyde oxidase inhibitor
**0.144**	0.029	Aldehyde dehydrogenase substrate
**0.217**	0.103	NOS2 expression inhibitor
**0.186**	0.074	Malate dehydrogenase inhibitor
**0.267**	0.155	Malate dehydrogenase (acceptor) inhibitor
**0.267**	0.156	GABA aminotransferase inhibitor
**0.148**	0.038	Aspartate kinase inhibitor
**0.158**	0.047	Cyclooxygenase inhibitor
**0.162**	0.052	3-Ketovalidoxylamine C-N-lyase inhibitor
**0.194**	0.085	Levanase inhibitor
**0.123**	0.014	Glutathione S-transferase inhibitor
**0.235**	0.128	4-Hydroxymandelate oxidase inhibitor
**0.177**	0.070	Glyoxylate reductase (NADP+) inhibitor
**0.231**	0.126	Urethanase inhibitor
**0.205**	0.099	Dopamine release stimulant
**0.212**	0.109	Long-chain-aldehyde dehydrogenase inhibitor
**0.176**	0.073	2,3-Dihydroxyindole 2,3-dioxygenase inhibitor
**0.224**	0.122	Transcription factor NF kappa A inhibitor
**0.177**	0.075	Aureolysin inhibitor
**0.181**	0.080	CYP4F2 substrate
**0.180**	0.080	3-Demethylubiquinone-9 3-O-methyltransferase inhibitor
**0.197**	0.097	Glutaminase inhibitor
**0.334**	0.235	Polyporopepsin inhibitor
**0.167**	0.067	Gametolysin inhibitor
**0.194**	0.095	Antiviral (Hepatitis B)
**0.281**	0.181	2-Dehydropantoate 2-reductase inhibitor
**0.107**	0.008	Prostaglandin agonist
**0.182**	0.084	Polygalacturonase inhibitor
**0.306**	0.209	Trans-acenaphthene-1,2-diol dehydrogenase inhibitor
**0.193**	0.096	Oryzin inhibitor
**0.299**	0.204	Fibrinolytic
**0.196**	0.101	Ceramide glucosyltransferase inhibitor
**0.195**	0.101	Thiopurine S-methyltransferase inhibitor
**0.136**	0.042	Pyrimidine-deoxynucleoside 2'-dioxygenase inhibitor
**0.251**	0.157	CYP2C9 substrate
**0.224**	0.130	Phosphopantothenoylcysteine decarboxylase inhibitor
**0.215**	0.122	Phospholipase C inhibitor

**0.244**	0.151	Antithrombotic
**0.142**	0.051	Thromboxane synthase stimulant
**0.128**	0.037	UDP-N-acetylglucosamine 1-carboxyvinyltransferase inhibitor
**0.213**	0.123	Flavin-containing monooxygenase substrate
**0.157**	0.068	Peptidylamidoglycolate lyase inhibitor
**0.181**	0.093	Phospholipase A1 inhibitor
**0.173**	0.087	Metallocarboxypeptidase D inhibitor
**0.211**	0.125	Angiogenesis stimulant
**0.136**	0.049	Calcium channel (voltage-sensitive) blocker
**0.187**	0.101	Peroxidase inhibitor
**0.172**	0.086	Polyneuridine-aldehyde esterase inhibitor
**0.199**	0.114	Antipyretic
**0.312**	0.228	Antiviral (Rhinovirus)
**0.254**	0.169	CYP3A5 substrate
**0.258**	0.174	Biotinidase inhibitor
**0.188**	0.106	UDP-glucuronosyltransferase substrate
**0.120**	0.039	tRNA (guanine-N1-)-methyltransferase inhibitor
**0.204**	0.123	Catenin beta inhibitor
**0.239**	0.158	Fatty-acyl-CoA synthase inhibitor
**0.224**	0.143	Arylsulfate sulfotransferase inhibitor
**0.158**	0.078	Alkylator
**0.252**	0.173	Gonadotropin antagonist
**0.152**	0.073	Thioredoxin reductase inhibitor
**0.159**	0.080	4-Hydroxybenzoate nonaprenyltransferase inhibitor
**0.133**	0.054	Kexin inhibitor
**0.188**	0.110	Diabetic nephropathy treatment
**0.133**	0.055	Furin inhibitor
**0.319**	0.241	Testosterone 17beta-dehydrogenase (NADP+) inhibitor
**0.227**	0.149	Antiinflammatory, intestinal
**0.158**	0.081	Licheninase inhibitor
**0.274**	0.197	UDP-N-acetylglucosamine 4-epimerase inhibitor
**0.151**	0.075	Shikimate 5-dehydrogenase inhibitor
**0.163**	0.088	Glycine C-acetyltransferase inhibitor
**0.235**	0.160	MMP9 expression inhibitor
**0.125**	0.050	Glycerol-3-phosphate dehydrogenase (NAD+) inhibitor
**0.286**	0.211	Gastrin inhibitor
**0.110**	0.036	Chelator
**0.239**	0.165	NAD(P)+-arginine ADP-ribosyltransferase inhibitor
**0.288**	0.214	Alopecia treatment
**0.151**	0.077	Homoserine dehydrogenase inhibitor
**0.087**	0.014	Leukotriene B4 antagonist
**0.188**	0.115	O-aminophenol oxidase inhibitor

**0.244**	0.171	Histidine N-acetyltransferase inhibitor
**0.097**	0.024	Dihydrodipicolinate synthase inhibitor
**0.142**	0.070	Ganglioside galactosyltransferase inhibitor
**0.170**	0.099	Tauropine dehydrogenase inhibitor
**0.178**	0.107	Antitussive
**0.217**	0.146	D-lactaldehyde dehydrogenase inhibitor
**0.196**	0.126	Saluretic
**0.181**	0.111	Cyclohexyl-isocyanide hydratase inhibitor
**0.185**	0.115	Calpain inhibitor
**0.251**	0.182	Nitrate reductase (cytochrome) inhibitor
**0.210**	0.141	CYP2A5 substrate
**0.148**	0.079	Procollagen C-endopeptidase inhibitor
**0.203**	0.134	Lysostaphin inhibitor
**0.140**	0.071	UGT1A7 substrate
**0.161**	0.093	Mannitol-1-phosphatase inhibitor
**0.274**	0.206	Mucositis treatment
**0.074**	0.006	Prostaglandin E2 antagonist
**0.160**	0.092	Nitric oxide scavenger
**0.185**	0.118	Nardilysin inhibitor
**0.199**	0.132	Glycerol-3-phosphate dehydrogenase inhibitor
**0.295**	0.228	Macrophage colony stimulating factor agonist
**0.124**	0.057	Keratoses actinic (solar) treatment
**0.261**	0.194	Glycerol-3-phosphate oxidase inhibitor
**0.254**	0.187	CYP2A1 substrate
**0.164**	0.097	N-(long-chain-acyl)ethanolamine deacylase inhibitor
**0.134**	0.068	Succinic dehydrogenase inhibitor
**0.197**	0.131	Antidiabetic symptomatic
**0.115**	0.050	Serine O-acetyltransferase inhibitor
**0.134**	0.069	Diisopropyl-fluorophosphatase inhibitor
**0.096**	0.031	Thromboxane antagonist
**0.154**	0.090	Chemopreventive
**0.146**	0.082	Lactaldehyde reductase inhibitor
**0.250**	0.186	Insulysin inhibitor
**0.213**	0.150	4-Methoxybenzoate monooxygenase (O-demethylating) inhibitor
**0.167**	0.104	Galactolipase inhibitor
**0.257**	0.195	Muramoyltetrapeptide carboxypeptidase inhibitor
**0.160**	0.098	Antiparkinsonian, tremor relieving
**0.135**	0.074	Cellulase inhibitor
**0.121**	0.061	Pectin lyase inhibitor
**0.125**	0.065	Neurotrophic factor
**0.078**	0.018	Cruzipain inhibitor
**0.207**	0.147	Hematopoietic inhibitor

**0.157**	0.098	ADP-ribosylarginine hydrolase inhibitor
**0.094**	0.034	High-mannose-oligosaccharide beta-1,4-N-acetylglucosaminyltransferase inhibitor
**0.229**	0.170	Formaldehyde transketolase inhibitor
**0.126**	0.067	Gelatinase inhibitor
**0.207**	0.149	Methylumbelliferyl-acetate deacetylase inhibitor
**0.257**	0.199	Lactase inhibitor
**0.224**	0.166	Glyoxylate reductase inhibitor
**0.253**	0.196	Calcium regulator
**0.176**	0.119	Proliferative diseases treatment
**0.239**	0.182	UGT2B12 substrate
**0.272**	0.215	Aminobutyraldehyde dehydrogenase inhibitor
**0.192**	0.136	Immunostimulant
**0.153**	0.097	Dynein ATPase inhibitor
**0.138**	0.083	Acylaminoacyl-peptidase inhibitor
**0.190**	0.134	Leukotriene-B4 20-monooxygenase inhibitor
**0.134**	0.078	Glucan 1,4-beta-glucosidase inhibitor
**0.072**	0.017	Prostaglandin antagonist
**0.378**	0.323	Gluconate 2-dehydrogenase (acceptor) inhibitor
**0.078**	0.023	Leukotriene C antagonist
**0.166**	0.111	Xylan endo-1,3-beta-xylosidase inhibitor
**0.206**	0.152	N-benzyloxycarbonylglycine hydrolase inhibitor
**0.250**	0.197	Beta-adrenergic receptor kinase inhibitor
**0.250**	0.197	G-protein-coupled receptor kinase inhibitor
**0.111**	0.058	Squalene epoxidase inhibitor
**0.241**	0.188	CYP3A1 substrate
**0.115**	0.062	Glycerol-3-phosphate O-acyltransferase inhibitor
**0.059**	0.007	Carbonic anhydrase XIV inhibitor
**0.135**	0.082	Tripeptide aminopeptidase inhibitor
**0.185**	0.134	Coccolysin inhibitor
**0.074**	0.023	Peroxisome proliferator-activated receptor agonist
**0.121**	0.070	Xanthine oxidase inhibitor
**0.157**	0.106	Alkylglycerophosphoethanolamine phosphodiesterase inhibitor
**0.242**	0.191	Aspartate-phenylpyruvate transaminase inhibitor
**0.228**	0.178	CYP2C substrate
**0.134**	0.084	Magnesium-protoporphyrin IX methyltransferase inhibitor
**0.235**	0.186	Cyanoalanine nitrilase inhibitor
**0.129**	0.080	Interleukin 1 antagonist
**0.181**	0.132	Glucan 1,4-alpha-maltotetraohydrolase inhibitor
**0.069**	0.020	Vanilloid 4 antagonist
**0.127**	0.079	Acylglycerone-phosphate reductase inhibitor
**0.055**	0.006	VCAM-1 antagonist
**0.204**	0.156	CYP2C18 substrate

**0.054**	0.007	VCAM antagonist
**0.151**	0.104	Myeloblastin inhibitor
**0.077**	0.030	SULT1A2 substrate
**0.083**	0.036	Long-chain-fatty-acid-CoA ligase inhibitor
**0.254**	0.207	CF transmembrane conductance regulator agonist
**0.069**	0.023	Homoserine O-acetyltransferase inhibitor
**0.116**	0.069	Glycerone-phosphate O-acyltransferase inhibitor
**0.067**	0.021	Retinoid X alpha receptor agonist
**0.114**	0.068	Monophenol monooxygenase inhibitor
**0.107**	0.062	Pantothenase inhibitor
**0.119**	0.074	Adenylate cyclase inhibitor
**0.090**	0.045	Ornithine decarboxylase inhibitor
**0.100**	0.056	CDC25A inhibitor
**0.246**	0.202	Carboxypeptidase Taq inhibitor
**0.169**	0.125	Antihelmintic
**0.072**	0.029	Antihypermotility
**0.091**	0.048	Protein-tyrosine phosphatase 1B inhibitor
**0.199**	0.157	NADPH-ferrihemoprotein reductase inhibitor
**0.158**	0.117	Mucorpepsin inhibitor
**0.124**	0.083	Interleukin 4 antagonist
**0.221**	0.180	Acetylesterase inhibitor
**0.117**	0.076	Malate-CoA ligase inhibitor
**0.085**	0.045	[acyl-carrier-protein] S-malonyltransferase inhibitor
**0.117**	0.077	N-(5-amino-5-carboxypentanoyl)-L-cysteinyl-D-valine synthase inhibitor
**0.053**	0.013	Carbonic anhydrase VII inhibitor
**0.333**	0.293	Nootropic
**0.159**	0.120	Dihydroxy-acid dehydratase inhibitor
**0.060**	0.021	Calcium channel L-type blocker
**0.185**	0.147	Platelet aggregation inhibitor
**0.231**	0.193	JAK2 expression inhibitor
**0.254**	0.216	Dementia treatment
**0.141**	0.103	1,4-Alpha-glucan branching enzyme inhibitor
**0.215**	0.178	Antiprotozoal (Leishmania)
**0.093**	0.056	Cytokine release inhibitor
**0.054**	0.017	Alpha-amino-acid esterase inhibitor
**0.068**	0.031	1-Alkyl-2-acetylglycerol O-acyltransferase inhibitor
**0.057**	0.020	Lysophosphatidic acid receptor antagonist
**0.224**	0.187	Histidine kinase inhibitor
**0.166**	0.129	PfA-M1 aminopeptidase inhibitor
**0.288**	0.251	Platelet aggregation stimulant
**0.177**	0.141	TRPA1 agonist
**0.257**	0.222	Caspase 3 stimulant

**0.124**	0.088	Cysteine synthase inhibitor
**0.202**	0.167	FMO1 substrate
**0.040**	0.005	Juvenile-hormone esterase inhibitor
**0.173**	0.139	Linoleoyl-CoA desaturase inhibitor
**0.102**	0.069	Palmitoyl-CoA hydrolase inhibitor
**0.102**	0.069	Acyl-CoA hydrolase inhibitor
**0.244**	0.210	CYP2C8 substrate
**0.126**	0.093	D-xylulose reductase inhibitor
**0.099**	0.066	Sorbose dehydrogenase inhibitor
**0.102**	0.070	Dimethylhistidine N-methyltransferase inhibitor
**0.180**	0.148	H+-exporting ATPase inhibitor
**0.137**	0.105	Styrene-oxide isomerase inhibitor
**0.253**	0.221	Pin1 inhibitor
**0.115**	0.084	Glycosylphosphatidylinositol diacylglycerol-lyase inhibitor
**0.053**	0.022	Peroxisome proliferator-activated receptor alpha agonist
**0.130**	0.098	NAD+ synthase (glutamine-hydrolysing) inhibitor
**0.256**	0.225	Sulfur reductase inhibitor
**0.073**	0.042	3-Dehydroquinate synthase inhibitor
**0.110**	0.079	Glucosamine-6-phosphate deaminase inhibitor
**0.116**	0.086	Bcl2 antagonist
**0.178**	0.148	Acetylserotonin O-methyltransferase inhibitor
**0.126**	0.096	Corticosteroid antagonist
**0.192**	0.162	Amyotrophic lateral sclerosis treatment
**0.288**	0.259	CYP2J2 substrate
**0.084**	0.055	Sporulation kinase A inhibitor
**0.184**	0.155	Thyroxine 5-deiodinase inhibitor
**0.143**	0.114	Sedoheptulose-bisphosphatase inhibitor
**0.099**	0.070	Phosphoglycerate mutase inhibitor
**0.199**	0.170	Hydroxylamine reductase (NADH) inhibitor
**0.186**	0.158	ADP-thymidine kinase inhibitor
**0.100**	0.072	Site-specific DNA-methyltransferase (adenine-specific) inhibitor
**0.085**	0.057	Interleukin 1b antagonist
**0.127**	0.099	IgA-specific metalloendopeptidase inhibitor
**0.088**	0.061	Pantetheine-phosphate adenylyltransferase inhibitor
**0.126**	0.098	Cysteamine dioxygenase inhibitor
**0.211**	0.184	Antineurogenic pain
**0.275**	0.249	Complement factor D inhibitor
**0.070**	0.044	Anesthetic inhalation
**0.224**	0.199	Glycogen synthase stimulant
**0.166**	0.141	Fructan beta-fructosidase inhibitor
**0.091**	0.066	Acyloxyacyl hydrolase inhibitor
**0.097**	0.073	Protein-tyrosine phosphatase inhibitor

**0.057**	0.033	CDC25B inhibitor
**0.198**	0.175	Venom exonuclease inhibitor
**0.263**	0.240	Neurotransmitter antagonist
**0.099**	0.076	Sulfotransferase substrate
**0.075**	0.053	Amino-acid racemase inhibitor
**0.089**	0.067	Arylformamidase inhibitor
**0.061**	0.039	Mannosidase inhibitor
**0.090**	0.068	4-Alpha-glucanotransferase inhibitor
**0.103**	0.081	Protein phosphatase inhibitor
**0.150**	0.128	Glycerol 2-dehydrogenase (NADP+) inhibitor
**0.124**	0.103	Sphinganine-1-phosphate aldolase inhibitor
**0.048**	0.026	tRNA (adenine-N1-)-methyltransferase inhibitor
**0.179**	0.158	Endothelial growth factor antagonist
**0.060**	0.040	N-acetylmuramoyl-L-alanine amidase inhibitor
**0.101**	0.081	NAD+ kinase inhibitor
**0.133**	0.113	N-methyl-2-oxoglutaramate hydrolase inhibitor
**0.043**	0.023	Leukotriene C4 antagonist
**0.186**	0.167	Antiischemic
**0.137**	0.118	Protein-Npi-phosphohistidine-sugar phosphotransferase inhibitor
**0.180**	0.161	Antiparkinsonian, rigidity relieving
**0.028**	0.010	Retinoic acid receptor agonist
**0.076**	0.057	mRNA (guanine-N7-)-methyltransferase inhibitor
**0.020**	0.002	Eukaryotic elongation factor 2 kinase inhibitor
**0.256**	0.238	2-Hydroxyquinoline 8-monooxygenase inhibitor
**0.144**	0.127	2-Oxoaldehyde dehydrogenase (NADP+) inhibitor
**0.144**	0.126	Prenyl-diphosphatase inhibitor
**0.058**	0.041	Angiotensin-converting enzyme inhibitor
**0.047**	0.030	Phospholipase A2 IIa inhibitor
**0.143**	0.127	Vitamin-K-epoxide reductase (warfarin-insensitive) inhibitor
**0.020**	0.004	Prostaglandin EP12 antagonist
**0.157**	0.141	S-alkylcysteine lyase inhibitor
**0.133**	0.117	[phosphorylase] phosphatase inhibitor
**0.069**	0.053	Pantoate-beta-alanine ligase inhibitor
**0.039**	0.023	Prostacyclin antagonist
**0.166**	0.151	Uroporphyrinogen-III synthase inhibitor
**0.183**	0.168	Vascular dementia treatment
**0.125**	0.111	HMG CoA synthase inhibitor
**0.099**	0.085	Asparaginase inhibitor
**0.195**	0.181	Dehydro-L-gulonate decarboxylase inhibitor
**0.177**	0.163	tRNA-pseudouridine synthase I inhibitor
**0.229**	0.216	Sulfite dehydrogenase inhibitor
**0.147**	0.134	Ketol-acid reductoisomerase inhibitor

**0.233**	0.219	3-Hydroxybenzoate 6-monooxygenase inhibitor
**0.036**	0.022	Leukotriene D4 antagonist
**0.201**	0.188	Adenylyl-sulfate reductase inhibitor
**0.193**	0.180	Exoribonuclease II inhibitor
**0.132**	0.119	Envelysin inhibitor
**0.165**	0.153	Antineoplastic enhancer
**0.051**	0.040	Magnolysin inhibitor
**0.182**	0.170	Glucan 1,4-alpha-maltotriohydrolase inhibitor
**0.161**	0.150	Glutathione dehydrogenase (ascorbate) inhibitor
**0.194**	0.184	Peptide-N4-(N-acetyl-beta-glucosaminyl)asparagine amidase inhibitor
**0.249**	0.239	Prion diseases treatment
**0.102**	0.092	Steroid 9alpha-monooxygenase inhibitor
**0.012**	0.003	Apolipoprotein A inhibitor
**0.252**	0.243	Rubredoxin-NAD+ reductase inhibitor
**0.183**	0.175	Peptide alpha-N-acetyltransferase inhibitor
**0.216**	0.207	Hydroxylamine oxidase inhibitor
**0.051**	0.042	D-Serine ammonia-lyase inhibitor
**0.071**	0.063	S-methyl-5-thioribose kinase inhibitor
**0.126**	0.117	L-glucuronate reductase inhibitor
**0.047**	0.039	Cysteine dioxygenase inhibitor
**0.046**	0.039	Secretory phospholipase A2 inhibitor
**0.012**	0.004	Factor IXa inhibitor
**0.033**	0.026	Dopamine precursors
**0.162**	0.155	Peroxidase substrate
**0.063**	0.057	L-3-cyanoalanine synthase inhibitor
**0.127**	0.121	Phosphatidylglycerophosphatase inhibitor
**0.224**	0.219	CYP3A substrate
**0.077**	0.071	Retinoic acid beta receptor agonist
**0.183**	0.177	AR expression inhibitor
**0.114**	0.109	Guanidinoacetate N-methyltransferase inhibitor
**0.154**	0.149	Sorbitol-6-phosphate 2-dehydrogenase inhibitor
**0.077**	0.072	Cyclooxygenase 2 inhibitor
**0.107**	0.102	Phosphoenolpyruvate-protein phosphotransferase inhibitor
**0.012**	0.007	Caspase 7 inhibitor
**0.284**	0.279	Glutamyl endopeptidase II inhibitor
**0.128**	0.124	Paraoxonase substrate
**0.112**	0.108	Farnesyltranstransferase inhibitor
**0.107**	0.103	Radical formation agonist
**0.042**	0.038	Elastase inhibitor
**0.040**	0.037	Acetyl-CoA transferase 2 inhibitor
**0.065**	0.062	Acetate-CoA ligase inhibitor
**0.100**	0.097	Beta lactamase inhibitor

**0.175**	0.173	Xenobiotic-transporting ATPase inhibitor
**0.087**	0.085	6-Methylsalicylate decarboxylase inhibitor
**0.114**	0.113	Phenol 2-monooxygenase inhibitor
**0.164**	0.163	1,2-alpha-L-fucosidase inhibitor
**0.079**	0.077	Glycine hydroxymethyltransferase inhibitor
**0.158**	0.157	Diuretic
**0.111**	0.110	Phenylacetate-CoA ligase inhibitor
**0.179**	0.178	Antihypertensive
**0.122**	0.120	Arylacetonitrilase inhibitor
**0.211**	0.210	Antiprotozoal (Amoeba)
**0.084**	0.083	Homocysteine desulfhydrase inhibitor
**0.190**	0.189	Gluconate 5-dehydrogenase inhibitor
**0.163**	0.163	3-Methylbutanal reductase inhibitor
